# Predictors of the number of under-five malnourished children in Bangladesh: application of the generalized poisson regression model

**DOI:** 10.1186/1471-2458-13-11

**Published:** 2013-01-08

**Authors:** Mohammad Mafijul Islam, Morshed Alam, Md Tariquzaman, Mohammad Alamgir Kabir, Rokhsona Pervin, Munni Begum, Md Mobarak Hossain Khan

**Affiliations:** 1 Department of Statistics, Jahangirnagar University, Savar, Dhaka, -1342, Bangladesh; 2 Department of Mathematics & Statistics, Bowling Green State University, Bowling Green, OH, 43402, USA; 3 Probationary Senior Officer, Pubali Bank Ltd, Dhaka, Bangladesh; 4 Department of Applied Statistics, University of Malaya, Kuala Lumpur, Malaysia; 5 Department of Agricultural Statistics, Sher-e Bangla Nagar Agricultural University, Sher-e Bangla Nagar, Dhaka, -1207, Bangladesh; 6 Department of Mathematical Sciences, Ball State University, Muncie, IN, 47306, USA; 7 Department of Public Health Medicine, School of Public Health, University of Bielefeld, Bielefeld, Germany

**Keywords:** Malnutrition, Under-five children, Predictors, Generalized Poisson regression model, Bangladesh

## Abstract

**Background:**

Malnutrition is one of the principal causes of child mortality in developing countries including Bangladesh. According to our knowledge, most of the available studies, that addressed the issue of malnutrition among under-five children, considered the categorical (dichotomous/polychotomous) outcome variables and applied logistic regression (binary/multinomial) to find their predictors. In this study malnutrition variable (i.e. outcome) is defined as the number of under-five malnourished children in a family, which is a non-negative count variable. The purposes of the study are (i) to demonstrate the applicability of the generalized Poisson regression (GPR) model as an alternative of other statistical methods and (ii) to find some predictors of this outcome variable.

**Methods:**

The data is extracted from the Bangladesh Demographic and Health Survey (BDHS) 2007. Briefly, this survey employs a nationally representative sample which is based on a two-stage stratified sample of households. A total of 4,460 under-five children is analysed using various statistical techniques namely Chi-square test and GPR model.

**Results:**

The GPR model (as compared to the standard Poisson regression and negative Binomial regression) is found to be justified to study the above-mentioned outcome variable because of its under-dispersion (variance < mean) property**.** Our study also identify several significant predictors of the outcome variable namely mother’s education, father’s education, wealth index, sanitation status, source of drinking water, and total number of children ever born to a woman.

**Conclusions:**

Consistencies of our findings in light of many other studies suggest that the GPR model is an ideal alternative of other statistical models to analyse the number of under-five malnourished children in a family. Strategies based on significant predictors may improve the nutritional status of children in Bangladesh.

## Background

Malnutrition among children is a major public health problem in developing countries including Bangladesh [1-10]. Globally children with moderate and severe acute malnutrition are approximately 60 million and 13 million respectively [2]. Between 8 and 11 million under-five children also die each year in the world [2,9]. More than 50% of these deaths are attributed to malnutrition, which are mostly preventable through economic development and public health measures [2]. Although Bangladesh has already achieved a remarkable progress in reducing child malnutrition from 68% in the late 1980s to 41% in 2007 [6,11] and under-five mortality [12], still malnutrition is a common problem in this country [13,14]. It is one of the countries with very high burden of malnutrition [5,14]. The underlying cause for 60% of the under-five deaths is malnutrition in Bangladesh [5].

Malnutrition among children is a critical problem because its effects are long lasting and go beyond childhood. It has both short- and long-term consequences [6,8,10]. For instance, malnourished as compared to non-malnourished children are physically, emotionally and intellectually less productive and suffer more from chronic illnesses and disabilities [6,15,16]. Malnutrition among children depends on complex interactions of various factors reflecting socio-demographic, environmental, reproductive, institutional, cultural, political and regional factors [3,4,6,13,14,17-19]. Already many studies have been conducted to find the predictors of malnutrition in Bangladesh and elsewhere [7,8,20-28].

Poverty is found to be strongly associated with child malnutrition [4]. Although the relationship between economic ability and malnutrition is complex, a number of studies have illustrated that children of poorer households tend to be more undernourished than children of wealthier ones [3,13,22,24-26,29]. Parental education is also identified as a strong precdictor of child malnutrition [14,28]. However, the association of maternal education is relatively stronger than parental education [14,20,29]. Other determinants of child malnutrition may include social deprivation [23], rural-urban place of residence [18,30], religions [31,32], number of children [1,18], source of drinking water [1] and toilet facility [3,33].

Most of the abovementioned studies, that addressed the topics of child malnutrition, used categorical outcome variables and applied logistic regression (multinomial/binary) [7,10,14,27-29,34] models to find the predictors of child malnutrition. For instance, Mashal et al. [27] used multivariable logistic regression to identify the risk factors of malnutrition among children in Afghanistan. Mueller et al. [34] applied logistic regression to study the relationship of malnutrition with morbidity and mortality among West African children. Ojiako et al (2009) used Tobit model to find the determinants of malnutrition among preschool children in Nigeria [19]. Sometimes we see studies which addressed non-negative outcome variables like number of children in a household [35,36] and number of accidents [37]. Recently different types of regression based on Poisson distribution namely standard Poisson regression model, negative binomial regression model and generalized Poisson regression (GPR) model have been used to model such kind of count variables [36-38]. However, applications of these models are based on certain assumptions. For instance, standard Poisson regression model assumes equal mean and variance of the dependent variable. In reality, often this equality assumption is not true because the variance could be higher than mean (over-dispersion property) or lower than mean (under-dispersion property). Ignorance of these properties may produce biased standard errors and inefficient estimates of regression parameters, although the estimates of the standard Poisson model are still consistent. The negative binomial regression model is more flexible than the standard Poisson model and is frequently used to analyse outcome variable with over-dispersion. The GPR model, on the other hand, can capture both over- and under-dispersion properties of the data, which make this model even more flexible [37]. However, a count variable like number of children in a household often shows under-dispersion property [35]. Our data also shows the under-dispersion property. Therefore, we applied the GPR model to our data. The first objective was to demonstrate the applicability of this model as an alternative to study the child malnutrition in Bangladesh. The second objective was to find some predictors of the count variable defined as the number of under-five malnourished children in a Bangladeshi family.

## Methods

### Data source

The data was extracted from the Bangladesh Demographic and Health Survey (BDHS) conducted in 2007. This survey employs a nationally representative sample which is based on a two-stage stratified sample of households. This type of survey generally provides information on basic national indicators of social development. The BDHS 2007 was a part of the global Demographic and Health Survey (DHS) programme [39]. The present study utilised the information of 4,460 under-five children aged 0 to 59 months for whom anthropometric measurements were available.

### Dependent variable

Researchers can define nutritional status of children differently. The nutritional status of a child is typically based on several measurements namely height, weight, sex and age of the child. Three commonly used measures for nutritional status are height-for-age, weight-for-height and weight-for-age [40]. These measures are then expressed as Z-scores from the median of the reference population. In our study, we use ‘weight-for-height’ because it can describe current nutritional status by linking body mass in relation to body length. It does not require the exact age information of the child, which is necessary for the ‘weight-for-age’ [39]. It can also track whether a child recently receives sufficient contents of nutrients to build and maintain bodyweight along with other factors such as genetic growth, environment, and disease burden on activity level [41]. To define child malnutrition, we followed the national report of Bangladesh [39] and the guidelines of the World Health Organization [41]. According to these reports, a child is malnourished if the Z-score is below -2 standard deviation (SD) from the median of the reference population. The dependent variable in this study was expressed as the number of under-five malnourished children in a Bangladeshi family.

### Covariates/predictor variables

We consider several covariates (Table 1) as predictors which are commonly reported in the nutritional studies of children. Some of them (categories are given in parentheses) are place of residence (urban, rural ), parental education (no education, 1-5 years education, 6-10 years education, 11+ years education), father’s occupation (professional, business, farmer, worker; where worker means skilled, semi-skilled, factory worker, and blue color service), toilet facility (yes or no), sources of drinking water (tube well water, piped water, others; where others mean dug well water, unprotected water, river or dam or lake or ponds or canal water, rainfall water), religion (Islam, others; where other religions include Hinduism, Buddhism, Christian, and unknown religions), access to media (yes, no), wealth index (lowest, second, middle, fourth, highest), total number of children ever born to a woman, and total number of the children died in a family. Two of these variables namely wealth index and access to media are composite variables. The wealth index is an asset-based index that reflects the relative socioeconomic status of the household and is widely used in low- and middle-income countries to quantify inequalities and to control the confounding effect of socioeconomic variables. It is based on the household ownership variables (e.g. car, refrigerator, television), housing characteristics (e.g. materials of the floor, roof, walls) and access to services (e.g. availability of electricity) [12]. Access to media, which is also a composite index, is based on three mass media variables namely whether they listen to ratio, watch television, and read newspaper or magazine. This is categorized into two groups, where ‘yes’ means respondents have access to at least one of these media and ‘no’ means no access to any of these media.

**Table 1:** Basic characteristics of the parents and households in Bangladesh based on BDHS 2007

**Predictors**	**n (%)**	**Mean**	**Predictors**	**n (%)**	**Mean**
Place of residence:			Sources of drinking water:		
Urban	1590 (35.70)		Piped water	299 (6.70)	
Rural	2870 (64.30)		Tube well water	3595 (80.60)	
Mother’s education:			Others	566 (12.70)	
No education	1136 (25.50)		Religion:		
1-5 years education	1371 (30.70)		Islam	4050 (90.81)	
6-10 years education	1566 (35.10)		Others	410 (9.19)	
11+ years education	385 (8.60)		Access to media:		
Father’s education:			Yes	919 (29.10)	
No education	1437 (32.20)		No	3127 (70.10)	
1-5 years education	1240 (27.80)		Wealth index:		
6-10 years education	1184 (26.50)		Lowest quintile	849 (19.00)	
11+ years education	594 (13.30)		Second quintile	901 (20.20)	
Father’s occupation:			Middle quintile	835 (18.80)	
Farmer	1118 (25.53		Fourth quintile	850 (19.00)	
Worker	2003 (45.75)		Highest quintile	1025 (23.00)	
Professional	201 (4.59)				
Business	1056 (24.12)		Total number of children ever born to a woman		2.67
Toilet facility:			Total number of children dead in a family		0.24
Yes	3286 (74.00)				
No	1153 (26.00)				

### GPR model

The generalized Poisson probability function of the number of malnourished children (*Y)* in a family can be written as

(1) $$\begin{array}{c} f\left(y,\mu ,\alpha \right)={\left(\frac{\mu }{1+\alpha \mu }\right)}^{y}\frac{{\left(1+\mathrm{\alpha y}\right)}^{y-1}}{y!}\exp\left(-\frac{\left(1+\mathrm{\alpha y}\right)}{\left(1+\alpha \mu \right)}\right),\\ \;\;y=0,1,2,\ldots \end{array}$$

The mean and variance of *Y* are given by *E*(*Y*_*i*_| *x*_*i*_) = *μ*_*i*_ and *V*(*Y*_*i*_| *x*_*i*_) = *μ*_*i*_(1 + *αμ*_*i*_)^2^, where the mean of the dependent variable is related to the explanatory variables through the link function *μ*_*i*_ = *μ*_*i*_(*x*_*i*_) = exp(*x*_*i*_*β*). In this link function, *x*_*i*_ is a *k -* 1 dimensional vector of covariates, *β* is a *k*-dimensional vector of regression parameters, and *α* is a dispersion parameter. The standard Poisson regression model is a special form of the generalized Poisson regression model. When *α* is equal to zero, the probability function of generalized Poisson random variable reduces to the Poisson probability function. The positive value of *α* in equation (i) indicates the over-dispersion, whereas the negative value of *α* indicates the under-dispersion property of the distribution.

For selecting the right type of Poisson regression model, it is necessary to check the existence of dispersion problem in the data. The moment estimators of the two parameters in the Poisson distribution given by Consul and Jain [42] are as follows:

(2) $$\hat{\mu }=\sqrt{\frac{{\bar{y}}^{3}}{s^{2}}}$$

And

(3) $$\alpha =1-\sqrt{\frac{\bar{y}}{s^{2}}}$$

Where □ and *s*^2^ are sample mean and variance respectively. The asymptotic variances of the moment estimators given by Shoukri [43] are:

(4) $$V\left(\hat{\mu }\right)\approx \frac{\hat{\mu }}{2n}\left[\hat{\mu }+\frac{2-2\hat{\alpha }+3{\hat{\alpha }}^{2}}{1-\hat{\alpha }}\right]\text{,}$$

And

(5) $$V\left(\hat{\alpha }\right)\approx \frac{1-\hat{\alpha }}{2n\hat{\mu }}\left[\hat{\mu }-\hat{\mu }\hat{\alpha }+2\hat{\alpha }+3{\hat{\mu }}^{2}\right]$$

The adequacy of the GPR model over the PR model is assessed by setting the following hypothesis

(6) $$H_{0}:\alpha =0$$

Versus

(7) $$H_{1}:\alpha \neq 0.$$

This test of hypothesis determines whether the dispersion parameter is statistically different from zero. The rejection of *H*_0_ recommends the use of the GPR model rather than the standard Poisson regression model. To perform the test, the asymptotically normal Wald type “Z” statistic defined as the ratio of the estimate of *α* to its standard error is used.

The estimation of regression coefficients *β* is obtained by the maximum likelihood approach. The log-likelihood functions of the GPR model is

(8) $$\begin{array}{c} \mathrm{Log}\left(L\left(\beta ,\alpha ;y\right)\right)=\sum_{i=1}^{n}\left[y_{i}\log\left(\frac{\mu_{i}}{1+\alpha \mu_{i}}\right)+\left(y_{i}-1\right)\log\left(1+\alpha y_{i}\right)\right)\\ -\left(\frac{\mu_{i}\left(1+\alpha y_{i}\right)}{1+\alpha \mu_{i}}-\log\left(y!\right)\right] \end{array}$$

Where

(9) $$\mu_{i}=\mu_{i}\left(x_{i}\right)=\exp\left(x_{i}\beta \right)$$

### Statistical analysis

Simple summary statistics (either as percentage for the categorical variables or mean for the continuous variables) are shown for selected socioeconomic predictors (Table 1). At the outset of analyses, sample mean and sample variance of the dependent variable are calculated in order to check whether it follows the standard Poisson regression model or GPR model. Then the *Z* test is performed to check whether the dispersion parameter significantly deviates from zero. Here the null hypothesis (*H*_0_ : *α* = 0) states that the value of dispersion parameter is zero. In contrast, a two-sided alternative hypothesis (H_1_) is used which indicates that the value of the dispersion parameter is unequal to zero. Bivariate analyses (based on Pearson Chi-square test) are performed to examine association between dependent variable and each of the selected predictors (Table 2). All significant predictors are then finally included into the GPR model. As the dependent variable is more appropriate for the GPR model because of its under-dispersion property, we applied this model to estimate the regression parameters (*β*) including ‘p’ values based on Wald Chi-square values. Finally, incidence rate ratio (IRR) and 95% confidence interval are calculated for each group of the categorical predictors (Table 3). The statistical software packages SAS 9 and SPSS 11.5 are used to extract the information from BDHS 2007, to recode the variables, and to perform univariate and bivariate analyses. Finally we used R 2.14.1 to estimate parameters of the GPR model.

**Table 2:** Bivariate associations between the number of under-five malnourished children in a family and different predictors in Bangladesh, 2007

**Characteristics**	***χ***^**2**^	***P***
Place of residence	55.36^*^	<0.001
Mother’s education	252.70^*^	<0.001
Father’s education	241.91^*^	<0.001
Father’s occupation	123.75^*^	<0.001
Wealth index	253.21^*^	<0.001
Sources of drinking water	34.45^*^	<0.001
Toilet facility	47.48^*^	<0.001
Religion	0.01	0.925
Access to media	14.49^*^	<0.001
Total number of children ever-born to a woman	79.55^*^	<0.001
Total number of children died in a family	30.78^*^	<0.001

**Table 3:** Results of the multivariable generalized Poisson regression analysis to study the number of under-five malnourished children in Bangladesh, 2007

**Predictors**	**Categories**	**Estimated regression coefficient (ß)**	***χ***^**2**^	**P-value**	**Estimated IRR**	**95% CI for IRR**
Place of residence:	Urban	0.06	0.44	0.507	1.07	0.88-1.28
	Rural^(r)^					
Mother’s education:	No education	0.33*	7.01	0.008	1.39	1.09-1.78
	1-5 years education	0.32*	6.81	0.009	1.37	1.08-1.74
	6- 10 years education	0.22	3.00	0.083	1.24	0.97-1.59
	11+ years education^(r)^					
Father’s education:	No education	0.29*	5.17	0.023	1.33	1.04-1.71
	1-5 years education	0.26*	3.92	0.048	1.30	1.00-1.68
	6- 10 years education	0.20	2.26	0.133	1.22	0.94-1.58
	11+ years education^(r)^					
Father’s occupation:	Farmer	0.39	2.45	0.118	1.48	0.91-2.43
	Worker	0.32	2.04	0.153	1.39	0.89-2.17
	Professional	0.11	0.14	0.705	1.12	0.63-1.97
	Business^(r)^					
Wealth index:	Lowest quintile	0.50*	22.90	<0.001	1.64	1.34-2.01
	Second quintile	0.41*	10.38	0.001	1.50	1.17-1.93
	Middle quintile	0.33*	12.46	<0.001	1.39	1.16-1.67
	Fourth quintile	0.22*	4.46	0.035	1.25	1.02-1.53
	Highest quintile^(r)^					
Sources of drinking water:	Piped water	−0.24	3.18	0.075	0.79	0.61-1.02
	Tubewell water	−0.34*	16.48	<0.001	0.71	0.60-0.84
	Others^(r)^					
Toilet facility:	No	0.36*	56.32	<0.001	1.43	1.30-1.56
	Yes^(r)^					
Access to media:	No	0.08	1.41	0.235	1.08	0.95-1.22
	Yes^(r)^					
Total number of children ever born to a woman		0.05*	11.04	0.001	1.06	1.02-1.09
Total number of children dead in a family		−0.03	0.16	0.688	0.97	0.83-1.13

Notes: ^(r)^ indicates the reference group in each category.

*p < 0.05.

## Results

The estimated mean $\left(\bar{Y}=0.626\right)$ and variance (*s*_*y*_^2^ = 0.369) of the outcome variable reveal the under-dispersion property of the data. In the total sample, 16.9 percent of the under-five children are malnourished. Table 1 provides descriptive statistics for all predictors. According to this table, illiteracy rate is lower among mothers of children (26 percent) as compared to their fathers (32 percent). In contrast, the rate of higher education is higher among fathers of children (13.3 percent) than their mothers (8.6 percent). Nearly 30 percent of the families have access to the mass media. About one-fourth of the families (26 percent) have no toilet facility. The mean number of children ever born to a woman is 2.67.

Table 2 shows the summary results of bivariate analyses between outcome and predictor variables. All the predictors except religion show significant associations with outcome variable.

The estimated value of the dispersion parameter (*α*) and its standard deviation from equation (i) are -0.30266 and 0.000439, respectively. Our Null hypothesis formulated as *H*_0_ : *α* = 0, (*Z*=-14.4405 and p < 0.05) is rejected at 5% level of significance.

According to the results of GPR analysis (Table 3), variables namely mother’s education, father’s education, wealth index, toilet/sanitation, sources of drinking water, as well as total number of children are statistically associated with the child malnutrition in a family. The incidence rate of children suffering from malnutrition is higher for mothers having no education (IRR=1.39; 95% CI=1.09-1.78) and 1-5 years education (IRR=1.37; 95% CI= 1.08-1.74) as compared to mothers with higher education. Similar results are also found for father’s education. The incidence rate of malnutrition among children is estimated to be nearly 1.64 times higher in the lowest quintile than the highest quintile. Children of middle and fourth quintiles also show higher incidence rate of malnutrition as compared to the children of highest quintile. The children who drink piped water and tubewell water are nearly 21% and 29% less likely to experience malnutrition than the children drinking other sources of water (such as dug well water, unprotected well, surface water, unprotected spring, river or dam or lake or ponds or canal, rain water, etc). Similarly, toilet facility is strongly associated with malnutrition status of children. A child from a family having no sanitary toilet facility has 1.43 times higher incidence rate of experiencing malnutrition than a child with toilet facility. The total number of children ever-born in a family and the malnourished children are also positively associated.

## Discussion

Our study demonstrates that the GPR model is an ideal alternative to study the malnutritional status of children defined as the number of under-five malnourished children in a family. This model is a good alternative because most of the results of this study are found to be consistent with the findings of many other studies [1,3,7,10,14,19,26,28,31,33,44,45].

According to the GPR model, the malnutritional status of children is insignificant between rural and urban areas. This finding is both consistent [14] and contradictory [18]. A similar study in Vietnam [18] reports higher level of malnutrition in rural areas than urban areas. Some possible causes according to this study are lack of economic, socio-cultural, healthcare and intuitional facilities in rural areas [18]. Like in Vietnam, rural areas of Bangladesh also suffer from limited infrastructure and facilities in terms of modern healthcare services, sanitation, education, electricity and economic facilities. Particularly health services are concentrated in urban areas than rural areas [12]. Although urban-rural disparity in terms of child malnutrition is negligible in Bangladesh, this is not the case for many other indicators. For instance, one recent study reports remarkable urban-urban disparities for antenatal care service, age at marriage, fertility and child mortality in Bangladesh [46]. Another study in Vietnam also reports higher age at marriage, smaller family size and lower mortality rate of children in urban areas as compared to rural areas. What are the reasons for this inconsistency? One of the possible reasons might be related to the adjustment of the GPR model by other potential predictors, namely household wealth and maternal education [30]. Inclusion of other predictors along with place of residence into the same model may reduce the strength of urban-rural nutritional disparity. However, further elaborative research is warranted in this regard.

Many studies suggest that mother education is linked with child health outcomes. The relationship of maternal education with child malnutrition is more demonstrable than paternal education, health service availability, and socioeconomic status [14,20,29,47]. However, some studies also show parental educational effects on child nutrition [21,48]. Our study finds a significant positive relationship between mother’s education and child nutrition. This result is consistent with many other studies [1,3,7,8,10,14,18,19,26,28,31,44,45,47,49]. Such a relationship could exist because maternal schooling is strongly associated with good child care and good health. Women with higher as compared to lower educational level are more likely to raise their family income, which helps the families to provide more quality diets and better healthcare to their children. Additionally, educated mothers can efficiently use limited household resources and available healthcare facilities, limit their family size, maintain better health promoting behaviours and provide healthcare to their children [18,47]. All these factors positively contribute to the child nutrition.

The relationship between economic inequality and children nutritional status is investigated by many studies [3,26,29,45,50]. Generally the greater degree of economic disparity is associated with higher mortality [51]. The relationship between economic disparity and malnutrition at the national level is not straightforward, because better economy at the national level does not necessarily mean better health care for all. Social and economic disparity in a country may differently influence the accessibility to food and healthcare services including the burden of disease. A number of studies have illustrated that children from poorer households are more likely to be malnourished than children from wealthier households [3,22,24,25]. Social deprivation is also linked with a child's nutritional status [23]. In Bangladesh the nutritional status of children differs in different economic classes [26], which can be attributed to the fact that rich families have more ability to allocate necessary resources for their children than poor families. Understandably allocation of more resources to their children improves their health conditions by reducing multiple health risks.

Our finding reveals a strong positive association between number of children ever born to a woman and the number of under-five malnourished children in a family. These results are consistent with the findings of other study [1,18]. Generally families with more children experience more economic strain for food consumption and hence they are more likely to suffer from poor nutritional status. In other words, inadequate allocation of household resources among many children may lead to the low nutritional status. Particularly poor families cannot fulfil the nutritional requirements of the children. Families with more children generally devote less time to take care of their children [18]. Because of negative impacts of higher fertility on nutritional status of children, increasing birth interval should be an important strategy to improve the nutritional status among the under-five children.

In Bangladesh men are generally the main earner of a family, although employment opportunities are increasing for women due to the flourishing garment sectors. Income of the family is strongly associated with the type of father’s occupation. Normally fathers with more prestigious job have higher income than fathers with low level jobs and therefore children from the higher income families should have better nutritional status. However, this is not the case in our study. The insignificant association of father’s occupation with the nutritional status of children can be explained by the lack of proper nutritional knowledge and confounding effect of education of parents. Like father’s occupation, religion does not play any significant role in explaining the nutritional status of the under-five children in Bangladesh.

In our study factors namely source of drinking water and type of toilet also show significant association with child malnutrition. Similar results are reported by Pongou et al [3]. These are plausible because access to safe drinking water and hygienic toilet are the pre-conditions for maintaining good hygiene and nutrition among children. The incidence of various water-borne illnesses can be reduced with the improved supply of drinking water [15]. Therefore increasing access to the safe drinking water and hygienic sanitation are important to improve the nutritional status among under-five children.

This study has several strengths. The use of nationally representative data is one of the important strengths. Our findings could be reliable because of the large sample. Application of the GPR model as an alternative of other methods is another strength. Inclusion of right predictors into the model based on previous studies also increases the strength of the study. However, this study is not free from limitation. Firstly, all inherent limitations associated with the cross-sectional data are also true here. Another limitation of the study is that the model does not include regional and cultural variables, which are also reported as significant predictors [13]. Sex of the children is also not included in the model. Finally some socioeconomic variables are strongly correlated with each other (e.g. wealth index and education), which may produce biased estimates because of multicollinearity [13]. Exclusion of important predictors due to non-availability may also alter our findings.

## Conclusions

Our study demonstrates that the GPR model is an appropriate model to identify predictors affecting the nutritional status of children in Bangladesh. Father’s and mother’s education, wealth index, source of drinking water of the household, toilet facility, and total number of children ever born to a woman are significantly associated with child malnutrition in Bangladesh. Various strategies are reported by many studies [4-6,11,13,44]. Increasing educational facilities for mothers and fathers can improve the child nutrition. Facilitating access to safe drinking water and sanitation for poor families is also necessary to improve the child nutrition. Since higher fertility (i.e. number of children ever born to a woman) has a negative impact on child nutrition, government should implement policies to limit family size by increasing birth space [44]. Comprehensive and concerted nutritional interventions such as exclusive breastfeeding, complementary feeding, supplementation of micronutrients to children and mother, hygiene interventions, and management of severe malnutrition are also needed to improve child nutrition [4,8,11]. Other strategies such as public transportation to carry food and relief programmes for the disadvantaged groups are important to reduce child malnutrition [13]. Addressing inequity and general deprivation and implementation of other health programmes are also necessary to reduce malnutrition among children [4]. We should keep in mind that adequate nutrition of children is a prerequisite to build a healthy and productive nation [11]. In addition, to achieve the millennium development goal of halving child undernutrition by 2015, Bangladesh needs to scale up target-oriented programmes such as poverty-reduction income generating interventions and improvement of public food transports for the poor population and disadvantaged regions [13].

## Competing interests

The authors have no competing interests arising from the publication of this article.

## Authors’ contributions

MI, MA and MAK conceptualized the research topic together with MMHK and drafted the manuscript. MT mainly performed the data analysis. RP significantly contributed to the writing process and interpretation. MB revised the article critically and provided further inputs. MMHK finally structured the manuscript, collected the references and edited extensively before finalization. All authors read and approved the final manuscript.

## Pre-publication history

The pre-publication history for this paper can be accessed here:

http://www.biomedcentral.com/1471-2458/13/11/prepub
